# Repurposing Antipsychotics for Cancer Treatment

**DOI:** 10.3390/biomedicines9121785

**Published:** 2021-11-28

**Authors:** Nikolaos Vlachos, Marios Lampros, Spyridon Voulgaris, George A. Alexiou

**Affiliations:** Department of Neurosurgery, University Hospital of Ioannina, St. Niarhou Avenue, 45500 Ioannina, Greece; nickosvlachos826@gmail.com (N.V.); marioslampros@gmail.com (M.L.); svoulg@otenet.gr (S.V.)

**Keywords:** cancer, antipsychotics, repurposing

## Abstract

Cancer is a leading cause of death worldwide, with approximately 19 million new cases each year. Lately, several novel chemotherapeutic drugs have been introduced, efficiently inhibiting tumor growth and proliferation. However, developing a new drug is a time- and money-consuming process, requiring around 1 billion dollars and nearly ten years, with only a minority of the initially effective anti-cancer drugs experimentally finally being efficient in human clinical trials. Drug repurposing for cancer treatment is an optimal alternative as the safety of these drugs has been previously tested, and thus, in case of successful preclinical studies, can be introduced faster and with a lower cost into phase 3 clinical trials. Antipsychotic drugs are associated with anti-cancer properties and, lately, there has been an increasing interest in their role in cancer treatment. In the present review, we discussed in detail the in-vitro and in-vivo properties of the most common typical and atypical antipsychotics, along with their mechanism of action.

## 1. Introduction

Cancer is a leading cause of death in the world, with around 19 million new cancer cases and 10 million cancer-related deaths in 2020. These numbers are expected to increase in the following years, with new cancer cases projected to reach 28 million worldwide in 2040 [1]. Although chemotherapy plays a pivotal role in cancer treatment, in many cancer types, despite the initial vulnerability to chemotherapy, cancer cells become drug-resistant over time through different mechanisms such as DNA mutations which lead to drug inhibition and degradation, thus necessitating new drug development [2]. However, drug development is associated with a cost of around 1 billion dollars and an estimated 10 years on average from the initial discovery to final market approval [3]. Repurposing drugs for a variety of diseases has emerged as a cost- and time-effective strategy compared with the traditional development of a new drug [4]. Antipsychotic drugs, which are classified into typical/first-generation and atypical/second-generation antipsychotics, have been in clinical practice since the mid-1950s and early 1990s, respectively; thus, their adverse effects such as extrapyramidal symptoms (parkinsonism, dystonia) in the typical antipsychotics, agranulocytosis (clozapine), and elevated serum prolactin levels (risperidone) are well-documented [5].

The antipsychotic effect of typical antipsychotics is primarily mediated through inhibition of the D2 dopamine receptors (DRD2) in the mesolimbic dopaminergic pathway, the main brain pathway hyperactivated in patients with positive psychotic symptoms such as hallucinations and delirium, while atypical antipsychotics work by inhibiting DRD2 and serotonin receptors, most commonly the 5-HT2A subtype [6]. Recently, there have been studies supporting decreased incidence of some types of cancer in patients with schizophrenia treated with antipsychotic drugs [7,8], suggesting a possible anticancer activity. In this review, we investigated the anticancer properties of antipsychotic drugs focusing mainly on experimental studies (in vitro and in vivo) and the presumptive mechanism of action behind the anti-proliferative effect of each drug.

## 2. Typical Antipsychotics

### 2.1. Haloperidol

Haloperidol is a typical antipsychotic medication of the butyrophenones class synthesized by Janssen pharmaceuticals and used for numerous psychiatric conditions such as acute psychosis, schizophrenia, bipolar disorder, Tourette syndrome, delirium, agitation, and hallucinations related to alcohol withdrawal [9,10]. The antipsychotic properties of haloperidol are attributed to the blockage of DRD2 in the mesolimbic dopaminergic pathway. However, haloperidol is a non-selective inhibitor for D2 receptors and also has antimuscarinic, anti-adrenergic (a1), and antihistaminic (H1) effects [10,11]. Except for its use as an antipsychotic drug, haloperidol has also been found, mainly at an experimental level, to have anti-neoplastic, anti-fungal, anti-viral, and immunomodulatory properties [12,13,14,15].

Regarding its antineoplastic properties, haloperidol has been found to induce autophagy, apoptosis, and cell cycle arrest [15,16]. In an in vitro study, Papadopoulos et al. observed that glioblastoma (GBM) U87, U251, and T-98 cell lines are sensitive to haloperidol. Additionally, they found that haloperidol induces G_2_/M cell inhibition, increases the percentage of cells found in sub G_0_/G_1_ phase, and promotes caspase-8 activation, the latter suggesting the induction of apoptosis. Except for haloperidol’s effect in the cell cycle progression, they observed that haloperidol decreases the expression of CD24, CD44 adhesion molecules, inhibits wound-generated cell migration, and enhances GBM cell death when combined with the alkylating agent temozolomide (TMZ) and radiotherapy [15]. The synergistic effect of haloperidol in the treatment of GBM with TMZ was also supported in another study [17]. In that in vitro study, the authors found that the combination of TMZ with haloperidol or risperidone enhanced the anti-tumor effect compared with TMZ alone. Specifically, the authors observed that the activation of DRD2 is essential for the proliferation of glioma cells and that the combination of TMZ with DRD2 significantly inhibits GBM cell mitotic activity, increases the induction of DNA double-strand breaks (DSBs), as evident by the formation of γH2aX (a marker of DSBs), and inhibits the autophagic flux caused by TMZ in GBM cells. Moreover, they found that dopamine receptor antagonists can inhibit the TMZ-induced extracellular signal-related kinase (ERK) activation, suggesting a potential link between ERK activation and TMZ-mediated autophagy [17]. 

In a recent study, He et al. examined the effect of DRD2 inhibition in patient-derived glioblastoma xenograft models. DRD2 was inhibited by using haloperidol or ONC201, a small novel molecule selectively blocking DRD2. Subsequently, the authors studied the effects of this inhibition in the expression profiles of epidermal growth factor receptor (EGFR) and DRD2, observing an anti-correlation between the expression levels of these two molecules, suggesting a compensatory EGFR-mediated hyperactivation of their shared downstream effector REK when DRD2 are blocked, and resulting to haloperidol resistance. The latter finding indicates that EGFR levels in patients with GBM could be a potential biomarker to predict the effect of DRD2 inhibition in future clinical trials [18]. Haloperidol and other antipsychotics have been found to inhibit the Sonic Hedgehog signaling (SHH) pathway in glial cells by downregulating the expression of GLI-1, a key transcription factor of the SHH signaling pathway, via a complex mechanism involving the overexpression of 7-dehydrocholesterol reductase (7-DHCR) [16,19].

Haloperidol has also been found to cause DNA demethylation in MIA PaCa-2 human pancreatic carcinoma cells. In this cell line, the expression of Dual-specificity phosphatase 6 (DUSP6), an ERK1/2-selective phosphatase, has been suppressed with intronic methylation. Kim et al. observed that haloperidol induces CpG demethylation of DUSP6 in a dose-dependent manner, and the effect of demethylation was similar to the one with the demethylated agent 5-azacytidine. The latter effect was not observed in pancreatic cell line PANC-1, where the DUSP6 transcription was not suppressed [20]. In another study, Jandaghi et al. found that the protein levels of DRD2 were significantly increased in patients with pancreatic adenocarcinoma, and the inhibition of these receptors with haloperidol in orthotopic xenograft mice models reduced neoplasm size and metastasis [21]. 

**Figure 1:**
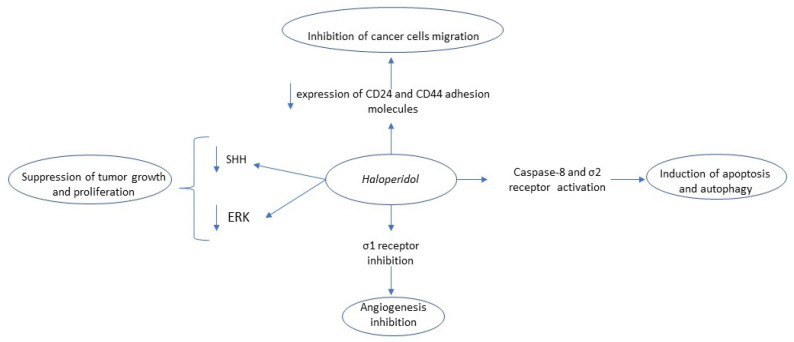
Summary of haloperidol’s anti-tumor mechanism of action. Key: SHH, Sonic hedgehog; ERK, extracellular signal-regulated kinase.

Sigma (σ) receptors are cell surface protein molecules found in multiple tissues and highly expressed in several neoplasms including prostate cancer, gliomas, breast cancer, and uveal melanoma [22,23]. Currently, two subtypes of σ receptors have been described, the σ1 a chaperon protein, found in mitochondria-associated membrane and involved in VEGF-mediated angiogenesis, and the σ2 receptor or ER-resident transmembrane protein 97, mediating cell proliferation and involved in apoptosis and autophagy. Even though their exact biologic role is currently unclear, there is mounting evidence that the inhibition of σ1 and simultaneous activation of σ2 have anti-tumor effects [22,23,24]. Additionally, the σ receptors decrease the expression of P-glycoprotein, a multidrug-resistant protein (MDR) that is an ATP-dependent efflux pump for several chemotherapeutic drugs. The haloperidol metabolite II is a molecule with such properties (σ1 antagonist/σ2 agonism) and its combination with 4-phenylbutyric acid (a Histone deacetylase inhibitor) in a single-ester molecule, the phenylbutyrate ester of haloperidol metabolite II (+/−MRJF4), or with valproic acid (+/− MRJF22) has been found to have an anti-tumor effect against prostate cancer cell lines (LNCaP, PC3) or rat C6 glioma cells [22,23,24], and uveal melanoma [25], respectively. The antitumor mechanisms of action of haloperidol are summarized in Figure 1.

### 2.2. Trifluoperazine

Trifluoperazine (TFP) is a commonly prescribed typical antipsychotic drug of the phenothiazines group, mainly utilized for schizophrenia treatment. However, the role of trifluoperazine as a potential anti-cancer agent is suggested by several authors [26,27]. TFP has been found to reduce the phenotype conversion of glioma cells into glioma initiating cells (GICs) by decreasing the radiation induced-Nanog expression, a pluripotency maintenance factor, and Glycogen synthase kinase-3 (GSK-3) activation. Moreover, the trifluoperazine decreased the number of GICs in GBM cell lines and increased survival in patient-derived orthotopic xenograft (PDOX) mouse models of GBM [27,28]. TFP has also been found to increase the radiosensitivity of GBM both in vitro and in vivo, by impairing homologous recombination and blocking autophagy by decreasing the levels of cathepsin L, a lysosomal cysteine protease [29]. In another study, Chen et al. found that trifluoperazine enhances the chemotherapeutic effect of doxorubicin in doxorubicin-resistant SHG44/DOX glioma cell lines by stimulating Forkhead box O1 (FOXO1) nuclear translocation. The FOXO1 is a transcription factor suppressing the expression of MDR genes, including P-gp, and its cytoplasmic translocation increases the expression of MDR genes, thus inducing chemotherapy resistance against various other agents such as gefitinib in lung cancer [30,31]. Regarding the role of TFP as an anti-glioma agent, Kang et al. reported that it can impair, in vitro and in vivo, intracellular calcium signaling by causing a massive and irreversible release from intracellular stores. Particularly, TFP binds to calmodulin subtype 2 (CaM2) and induces its releasement from the endoplasmic reticulum-Ca+2 channel- inositol 1,4,5-trisphosphate receptor (IP3R) subtype 3. The increased cytotoxic effect of the drug in glioma cells is probably explained by the increased expression levels of CaM2. Consequently, TFP induces impaired homeostasis of calcium intracellular signaling that inhibits glioma proliferation, invasion, and metastasis [32]. Its anti-metastatic activity was also noted by Pulkoski-Gross et al., who described a reduction in the levels of phosphorylated AKT (Ser473 and Thr308) and β-catenin (Ser552), induced by DRD2 blockage, ultimately leading to limited angiogenic (VEGF-mediated) and metastatic potential [33].

**Figure 2:**
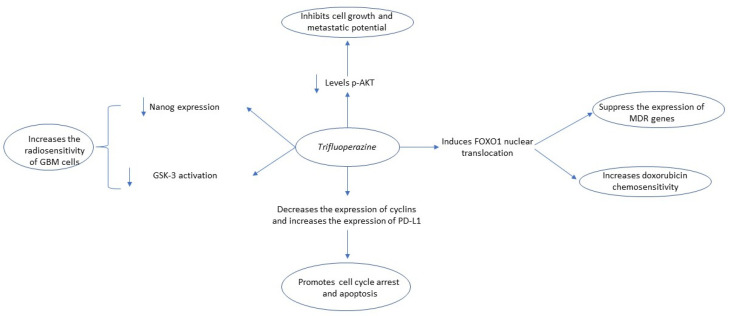
Summary of trifluoperazine’s anti-tumor mechanism of action. Key: GSK-3, Glycogen synthase kinase-3; p-AKT, Phosphorylated Akt, PD-L1, Programmed death-ligand 1; FOXO1, Forkhead Box O3; MDR, Multiple drug resistance; GBM, glioblastoma.

Currently, there is mounting evidence supporting that TFP can effectively induce G0/G1 cell cycle arrest and induce apoptosis [26,33,34]. Jiang et al. found that it can inhibit tumor growth in hepatocellular carcinoma (HCC) cell lines (SMMC-7721, Bel-7402), induce Go/G1 cell cycle arrest, and induce apoptosis through FOXO1 activation and increase of Bax to Bcl-2 ratio [35]. Except for its role in inducing cell cycle arrest and apoptosis in colorectal cancer (CRC), according to Xia et al., TFP increases the expression of programmed death-ligand (PD-L1), indicating that it can have a synergistic anti-cancer effect with immune checkpoint inhibitors [34]. Finally, Feng et al. observed that trifluoperazine impairs the expression of both cyclin D1/CDK4 and cyclin E/CDK2 in MDA-MB-468, MDA-MB-231, and 4T1 triple-negative breast cancer cell lines and increases survival of mice with brain metastasis [26,36]. The antitumor mechanisms of the action of trifluoperazine are summarized in Figure 2.

### 2.3. Chlorpromazine

Chlorpromazine is another typical antipsychotic drug of the phenothiazines class utilized for the treatment of schizophrenia. Several in vitro and in vivo studies have shown that chlorpromazine has multifaceted anti-cancer effects, including induction of apoptosis and inhibition of several intracellular signaling pathways and DNA synthesis [37,38]. Matteoni et al. reported that chlorpromazine had in vitro anti-tumor effects in six GBM lines, the anchorage-dependent cell lines T98G, U-251 MG, U-87 MG, and the anchorage-independent cell lines TS#1, TS#83, and TS#163 neurospheres. Chlorpromazine could inhibit cell viability, cause hyperdiploidy, reduce cloning, and downregulate the expression of stemness genes such as SOX2, NANOG, Nestin, and OLIG2. Additionally, chlorpromazine exhibited a synergistic effect with TMZ in the reduction of cell cloning [39] as well as with nitrosoureas in rat GBM cell lines (RG2) [40]. In another in vitro study, chlorpromazine inhibited the proliferation of TMZ-resistant glioma cell lines and glioma stem cells by inhibiting the CcO subunit 4 isoform 1 (COX4-1), which is mainly expressed in chemotherapy-resistant glioma cell lines [41]. In rat C6 glioma cell lines, chlorpromazine resulted in cell cycle arrest and an increase of p21Waf1/Cip1 level, a cyclin E/CDK2 inhibitor, by p53-independent induction of the transcription factor early growth response-1 (Egr-1) [42]. In U87MG glioma cell lines, chlorpromazine also induces autophagic cell death, but not apoptosis, through inhibiting the Akt/mTOR pathway [43]. A similar effect of chlorpromazine was observed in human oral cancer HSC-3 and Ca9-22 cell lines [44]. Kurita et al. found that chlorpromazine inhibits the interaction between RE1-silencing transcription factor/neuron-restrictive silencer factor (REST/NRSF), a transcription repressor overexpressed in medulloblastoma, and the paired amphipathic helix domains (PAH1) domain of mSin3 thus inhibiting the in vitro growth of medulloblastoma DAOY cell lines [45]. 

**Figure 3:**
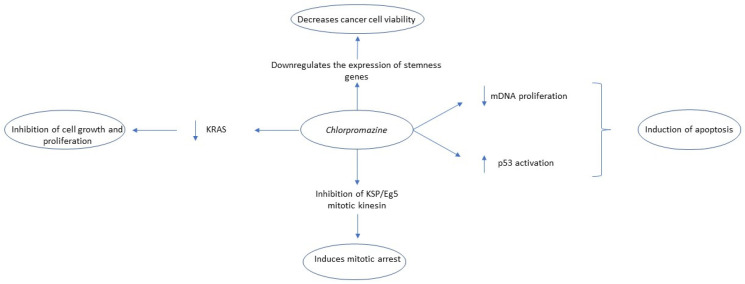
Summary of chlorpromazine’s anti-tumor mechanism of action. Key: KSP, Kinesin Spindle Protein; mDNA, mitochondrial DNA.

Regarding the anti-tumor effect of chlorpromazine in white blood cell malignancies, chlorpromazine appears to inhibit mitochondrial DNA polymerase and induce apoptosis selectively in several leukemia types, including acute T and acute B lymphoblastic leukemia, and Burkitt lymphoma cell lines but not in normal lymphocytes [46]. In a recent study, Rai et al. found that chlorpromazine caused the reduction, both in vitro and in vivo, in the levels of clathrin assembly lymphoid myeloid leukemia (CALM) protein and disturbed the subcellular localization of acute myeloid leukemia (AML)-mutated receptor tyrosine kinases (RTKs) such as FLT3-ITD and KIT-D816V, while sparing the non-mutated RTKs [47]. Similarly, in Ewing sarcoma, chlorpromazine impairs the clathrin mediated-insulin-like growth factor receptor 1 (IGFR1) internalization, involved in Ewing sarcoma pathogenesis, consequently decreasing proliferation rate and inducing apoptosis [48,49]. In diffuse large B-cell lymphoma, chlorpromazine promotes the expression of Sphingosine-1-phosphate receptor 2 (S1PR2), which is a G-coupled protein receptor involved in the maturation of B cells [50]. Chlorpromazine can also induce apoptosis in human colorectal cancer cell lines in a p53-dependent mechanism. Specifically, chlorpromazine induced p53 expression in CRC cell lines via activation of c-jun N-terminal kinase (JNK). The JNK promotes the degradation of sirtuin 1 (SIRT1), a histone deacetylase, and thus can block the deacetylation of p53, and its consequent deactivation [51]. The combination of chlorpromazine with pentamidine, an antiparasitic agent, seems to have a synergistic antiproliferative effect on non-small-cell lung carcinoma (NSCLC) cell line A549 in vitro and in vivo, as well as in human mice models xenografted with HCT116 colorectal carcinoma (CRC) cell line. Mechanistically, chlorpromazine inhibits mitotic kinesin KSP/Eg5 and induces mitotic arrest, while pentamidine impairs chromosome segregation [49]. The mutation of oncogenic Kirsten rat sarcoma virus (KRAS), a GTPase protein activating several key pathways involved in cell proliferation, survival, and transformation, is a major event involved in the pathogenesis of pancreatic cancer [52]. The anchoring of KRAS with the plasma membrane is necessary for the activation of KRAS-downstream signaling targets. Chlorpromazine was found to impair the association of oncogenic mutant (G12V) KRAS with the plasma membrane by modifying the electrostatic interactions of the KRAS- polybasic region with the plasma membrane, thus inducing cell cycle arrest and inhibition of cell growth and migration in PANC-1 cells [53]. Similarly, Yde et al. reported that chlorpromazine intercalates into hydrophobic regions of cell membranes and binds into negatively charged membrane surfaces, thus causing alterations in membrane permeability. Particularly, they observed that chlorpromazine had a synergistic effect with TMZ in reducing tamoxifen-resistant MCF-7/TAMR-1 breast cancer line growth by increasing tamoxifen’s cellular influx, probably through chlorpromazine-induced increased membrane permeability [54]. Finally, chlorpromazine suppressed the HIPPO signaling pathway, involved in cancer cell stemness and invasion, in a dose-dependent manner in the MCF-7 cell line, presumably by promoting proteasomal degradation, and decreased the chemotherapy resistance of cancer stem cells (CSC) [55]. The antitumor mechanisms of the action of chlorpromazine are summarized in Figure 3.

### 2.4. Pimozide

Pimozide is a first-generation antipsychotic drug of the diphenylbutylpiperidines class mainly utilized for treating schizophrenia and Gilles de la Tourette disease. Numerous preclinical studies have shown that pimozide has multifaceted anti-cancer effects such as inhibition of cell cycle and proliferation, induction of apoptosis, and cell invasion by interacting with multiple biological targets [56]. Recently, Hongo et al., through in-silico drug screening, found that pimozide inhibits aurora B kinase (AURKB) and kinesin family member 20 A (KIF20A), involved in spindle formation and cytokinesis, in castration-resistant DU145CR prostate cancer cell line, and its combination with taxane cabazitaxel appears to have a synergistic effect in the same line in vivo and in vitro [57]. In another study about the role of pimozide in prostate cancer treatment, pimozide reduced cell proliferation and migration by increasing the formation of reactive oxygen species (ROS) in PC3 and DU145 prostate cancer cell lines in vitro and in vivo in TRAMP mice [58], while in the study of Cai et al., pimozide had a similar effect in osteosarcoma U2OS cell lines by downregulating the expression of catalase, probably mediated through inhibition of the activity of signal transducer and activator of transcription 3 (STAT3) [59]. 

The STAT protein family encompasses transcription factors which are activated by Janus Kinases (JAK) and regulate the expression of oncogenes, the progression of cell cycle, and the induction of apoptosis. In triple-negative breast cancer cell lines, pimozide inhibited the STAT-3 mediated activation of matrix metalloproteinase-9 (MMP-9) and vimentin [60], while in brain tumor cell lines, it induced a STAT-3 mediated apoptosis in vitro and in vivo [61]. However, in another study, Dakir et al. reported that pimozide induced apoptosis in MDA-MB-231 breast cancer cells by downregulating the expression of Akt [62], while according to Strobl et al., the anti-tumor effect in breast cancer could relate to the calmodulin antagonist properties of pimozide [63]. Additionally, pimozide has been found to inhibit the in vitro phosphorylation of STAT5 in BCR-ABL positive and pSTAT5 overexpressing K562 chronic lymphocytic leukemia (CLL) cell lines [64,65], as well as in mice models with acute myeloid leukemia that contain the FLT3 internal tandem duplication (ITD) mutation [66]. A similar anti-cancer effect mediated by the inhibition of STAT5 phosphorylation was observed in Rat prolactinoma MMQ cells through inhibition of the STAT5/Bcl-xL and STAT5/cyclin D1 signaling pathways, in peripheral T-lymphoma through induction of the TRAIL/DR4-dependent apoptosis [67], and in osteosarcoma cells through inhibition of the proliferation of osteosarcoma stem cells [68]. Finally, Fako et al. found that haloperidol inhibits the in vitro growth of hepatocellular carcinoma Hep3B and HepG2 cell lines through impairment of the Wnt/β-catenin signaling pathway and its downstream molecules, including EpCAM, which is involved in cancer cell stemness [69].

### 2.5. Fluspirilene

Fluspirilene (FSP) is an antiphychotic drug that belongs to the disphenylbutylpiperidines class. Its main indication is schizophrenia treatment, antagonizing mainly the DRD2 and also calcium channels [70]. Preliminary data suggest FSP’s antitumor activity, both in vitro and in vivo in different cancer cells. Shi et al., in their in vitro studies, found that FSP attenuated viability and proliferation of HCC (HepG2, Huh cells) cell lines by reducing the expression of CDK2, Rb, pho-CDK2, pho-Rb, and cyclin E, all responsible for the transition from the G1 to S phase of the cell cycle, thus arresting the cancerous cells in G2 phase and subsequently inducing apoptosis. These findings were also validated in vivo where FSP reduced tumor size and volume in mice xenografted with Huh cells. The latter antineoplastic effect was comparable to that of 5-fluorouracil, the standard of care drug in HCC. Additionally, the combination of those two drugs provided the highest antitumor activity, suggesting a potential synergistic action [71]. In another study, Dong et al. described that FSP inhibited GBM’s increased mitotic activity and invasiveness in vitro by reducing the activity of signal transducer and activator of STAT-3, a pivotal transcription factor contributing to increased cell proliferation as well as treatment resistance. Of note, the inhibition of STAT-3 was observed in both glioma stem cells and differentiated glioma cells. Subsequently, the drug was tested in vivo, leading to significant tumor reduction (*p* = 0.017) and life prolongation (*p* = 0.026) in mice xenografted with cells with similar characteristics to GBM cells [72]. Finally, Patil et al., based on their own in vitro studies, proposed that FSP acts as a p53-MDM2 inhibitor by binding to the p-53 binding pocket of the MDM2 protein, causing activation of the tumor suppressor protein p53 and subsequent inhibition of tumor growth in human colon cells and several other human tumor cell lines in the NCI60 cell line panel [73].

### 2.6. Penfluridol

**Figure 4:**
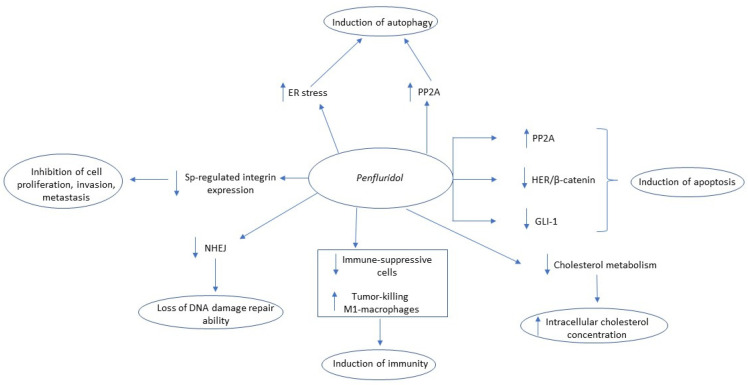
Summary of penfluridol’s anti-tumor mechanism of action. Key: PP2A, Protein phosphatase 2; NHEJ, Non-homologous end joining; ER, Endoplasmic reticulum; HER, Human epidermal growth factor receptor; GLI Family Zinc Finger 1, GLI-1.

Penfluridol represents a first-generation antipsychotic drug used for treating chronic schizophrenia and other psychotic disorders. Developed in the late 1960s at Janssen Pharmaceutical, penfluridol’s antipsychotic properties result from the blockade of dopamine receptors, particularly the D2 receptors. However, the drug also inhibits other dopamine receptors (D1, D3, D5) as well as T-type calcium-channel and 5HT2B receptors [74,75,76]. Recently, penfluridol has emerged as a potential anticancer drug, showing promising results both in vitro and in vivo in different types of cancer. Ranjan et al., in their in vitro studies with three triple-negative cancer cell lines (MDA-MB-231, HCC1806, 4T1), reported that penfluridol attenuated not only the expression of integrin α6, integrin β4, integrin α4, integrin α5, integrin β1, and integrin β3 but also integrin axis’ downstream molecules including FAK, p-Paxillin (Y118), and paxillin, ultimately inhibiting cell proliferation, invasiveness, and migration [77]. The integrin signaling pathway has been described as a key factor in tumor initiation, progression, and extravasation in secondary sites [78]. After orthotopic injection of 4T1 cells (in vivo), the same authors reported a 49% tumor volume reduction in penfluridol-treated mice, while after intracardiac and intracranial injections of the same cell line, the growth of metastatic brain tumors were suppressed by 90% and 72%, respectively, after penfluridol administration. The mechanism behind the tumor reduction was the one described in their in vitro study [77]. Based on this study, Hedrick et al. attempted to elucidate the molecular mechanism of integrins’ repression. The authors utilized two breast cancer cell lines (SKBR3, MDA-MB-231) for their in vitro experiments and found that penfluridol promotes ROS formation in both cell lines, which subsequently attenuates the expression of Specificity Factors (Sp) 1,3,4 and induces cleavage of PARP/caspase-3 (induction of apoptosis) [79]. Particularly, integrin expression is regulated by Sp transcriptional factors and orphan nuclear receptor 4A1 (NR4A1) [80,81]. Penfluridol does not antagonize NR4A1 but inhibits the expression of Sp 1, Sp 3, and Sp 4 (and therefore integrin expression) by downregulating ROS-dependent epigenetic expression of cMyc, decreased expression of cMyc-regulated miR-27a and miR-20a/miR-17, and induction of the Sp repressors ZBTB10 and ZBTB4 [79]. Another in vitro study investigated the effects of penfluridol in six different pancreatic cell lines (Panc1, Panc0504, Panc0403, SU8686, MiaPaCa2, AsPc1) and observed that the drug promoted the activity of protein phosphatase 2A (PP2A), an established a tumor-suppressor protein, leading to the suppression of phosphorylation of AKT, p70S6K, GSK3β and to MYC ubiquitination and degradation, and ultimately to cell death. Additionally, in the penfluridol-sensitive cells, there was increased expression of pro-apoptotic molecules (BIM, BAX, PUMA) and, simultaneously, decreased expression of anti-apoptotic proteins (e.g., Bcl-2), indicating an apoptotic-mediated pathway of cell death [82]. Activation of PP2A was also implicated as penfluridol’s mechanism of action in reducing the viability of AML cells (HL-60, U937, MV4-11) in vitro. The authors suggested that PP2A activation led to downregulation of Akt, ERK, and JNK signals and subsequently triggered caspase-mediated cellular apoptosis. The authors also observed high levels of ROS, but in contrast to Hendick et al., ROS appeared to play a protective role in the penfluridol-induced apoptosis [83]. In another study, penfluridol suppressed GBM cell in vitro proliferation by inhibiting the phosphorylation of Akt at Ser473, thus attenuating the expression of Akt-associated GLI-1, a highly expressed transcription factor in GBM cells, partly responsible for GBM’s treatment resistance as well as the expression of GLI1-mediated stem cells markers including OCT4, Nanog, and Sox2. Additionally, the drug-enhanced cleavage of caspase-3 and PARP indicated the promotion of cellular apoptosis. These results were confirmed in vivo, where penfluridol reduced the growth of GBM tumors in mice models after subcutaneous and intracranial injection by 65% and 72%, respectively [84]. The same molecule (GLI-1) was penfluridol’s target, according to Kim et al. [85]. The authors investigated the anticancer activity of penfluridol in glioma sphere-forming cell lines (GSCs) in vitro, a subpopulation of GBM cells responsible for treatment resistance and GBM recurrence, and reported an inhibitory effect of penfluridol on GLI-1, which subsequently reduced the GLI-1-associated markers of stemness (SOX2, NESTIN, OCT4), markers of invasion (Integrin α6, uPAR), and epithelial-mesenchymal transition (EMT) factors (Vimentin, Zeb-1, N-cadherin, Snail, Slug), molecules strongly associated with increased cell motility and resistance to genotoxic agents of GSCs. These findings were confirmed in orthotopic xenograft mice models (in vivo) where penfluridol-treated mice had a 30% tumor volume decrease compared with control with reduced expression of GSC-related factors, including GLI1, SOX2, and vimentin. This percentage surged to 80% in mice treated with the combination of temozolomide and penfluridol, suggesting a potential synergistic effect of the two drugs [85]. Another suggested mechanism of penfluridol’s cytotoxicity in pancreatic cancer, both in vitro (Panc-1, AsPC-1, BxPC-3) and in vivo, was the induction of autophagy-mediated apoptosis as indicated by increased cleavage of caspase-3 and PARP. At the same time, there was an increased conversion of LC3-I to LC3-II (a specific indicator of autophagosome formation) and high levels of p62, an autophagy-specific substrate, indicating that the increased autophagosome formation is accompanied by autophagosome-reduced degradation by lysosomes [86]. Penfluridol upregulated the expression of endoplasmic reticulum stress markers, including binding protein (BIP), C/EBP homologous protein (CHOP), and inositol requiring 1a (IRE1a), implicating the penfluridol-mediated endoplasmic reticulum system stress as the molecular mechanism behind the induction of autophagy which subsequently led to apoptosis [87]. The above findings were also confirmed in vitro [86,87]. The induction of autophagy by penfluridol, as indicated by increased levels of LC3-II and p62, was also observed in another in vitro study in NSCLC cell lines (A549, HCC827). The authors described that penfluridol promotes ER stress-mediated unfolded protein response (UPR) signaling pathways and activation of p38 mitogen-activated protein kinase (MAPK), both causing autophagosome accumulation in NSCLC cells [88]. However, in contrast to [86,87], given that autophagy is a major energy supplier for the cancer cell [89], the increased autophagosome synthesis promotes cell death by energy (ATP) depletion rather than apoptosis [88]. Another penfluridol-mediated inhibition of GBM cell (U87MG) proliferation in vivo was reported by Ranjan et al., describing a reduction of immune suppressive cells (T regulatory cells, myeloid-derived suppressive cells), upregulation of tumor-killing M1 macrophages, and decreased chronic inflammation biomarkers (CC14, IFNγ) in mice subcutaneously injected with U87MG GBM cells [90]. Gupta et al., using breast cancer cell lines (MCF-7, 4T1) in vitro and in vivo, found that penfluridol inhibits cell proliferation and migration by suppressing the HER2/β-catenin signaling pathway as well as its associated molecules (TCF-4, TCF-1, Cyclin D1, C-MYC, p-GSK3β) and inducing apoptosis (increased cleavage of caspase-3 and C1-PARP). Since this pathway is implicated in breast cancer paclitaxel (chemotherapeutic drug) resistance, its inhibition led to enhanced paclitaxel efficacy and significant tumor growth suppression (*p* = 0.0012) compared with controls in orthotopic mouse models (in vivo) [91]. Non-homologous end joining (NHEJ) is a mechanism of repair of ionizing radiation-induced double-stranded DNA breaks. Penfluridol-treated HeLa cells (in vitro), after 8Gy radiation, showed increased levels of broken DNA and loss of DNA damage repair processes, which were thought to be the result of inhibition of DNA-PKcs activation [92]. Finally, penfluridol inhibited the proliferation of melanoma (B16/F10), lung carcinoma (LL/2), and breast cancer (4T1) cell lines, both in vitro and in vivo, causing accumulation of unesterified cholesterol in all cancer cells. However, the exact mechanism of penfluridol-induced cholesterol dysregulation was not elucidated [93]. The antitumor mechanisms of action of penfluridol are summarized in Figure 4.

### 2.7. Thioridazine (THD)

**Figure 5:**
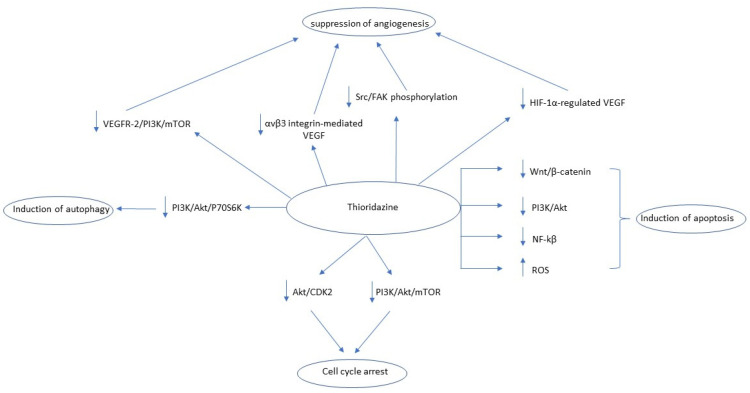
Summary of thioridazine’s anti-tumor mechanism of action. Key: VEGF, Vascular endothelial growth factor; PI3/AKT, phosphoinositide-3-kinase–protein kinase B/Akt HIF-1a, Hypoxia-inducible factor-1a; WNT, Wingless and Int-1; mTOR, Mammalian target of rapamycin; CDK2, Cyclin-dependent kinase 2; FAK, Focal adhesion kinase; ROS, Reactive oxygen species.

Thioridazine (THD) is a first-generation antipsychotic drug that belongs to the phenothiazines drug family, primarily blocking dopamine receptor 2 (DRD2), mainly used for the treatment of a wide range of psychotic disorders including schizophrenia and psychosis [94]. In cases of advanced cancer, THD may alleviate cancer-related sweating [95,96] and depression [97]. Regarding its potential anticancer effects, THD is one of the most studied antipsychotic drugs, being tested in various cancer cells, both in vitro and in vivo, including GBM cells [98,99,100,101,102], ovarian cancer cells [103,104,105,106,107], breast cancer cells [63,102,108,109,110], lung cancer cells [104,111], cervical cancer cells [112,113], endometrial cancer cells [112,114], melanoma cells [115,116], malignant blood cells (lymphoma, leukemia) [46,117,118,119], gastric cancer cells [120], malignant testicular germ cells [121], hepatocellular carcinoma cells [122], head and neck cancer cells [102], renal cancer cells [123], prostate cancer cells [124], neuroblastoma cells [101], and different types of cancer stem cells [125,126,127,128]. According to these studies, THD exerts its antiproliferative properties through discrepant molecular mechanisms. One of these mechanisms is the THD-induced cell cycle arrest, mainly in the G1 phase [98,103,104,105,108,109,111,112,120,122,125,126], DRD2 independently [109], through downregulation of the Akt/CDK2 pathway [125] or PI3K/Akt/mTOR/p70S6K pathway [105,112]. Another mechanism is the induction of the caspase- or mitochondrial-mediated apoptosis [46,98,100,101,102,103,104,105,107,108,110,111,112,113,114,115,116,117,118,120,123,124,125,127], which is achieved through inhibition of different pathways including the Wnt/β-catenin [98], PI3K/Akt [100,105,108,112,116], and NF-kβ pathways [110,118], while in some studies the apoptosis was ROS-mediated [102,107,108,123]. However, some studies reported that the THD’s antiproliferative effect was independent of apoptotic cell death induction [99,122]. A third mechanism was the THD-promoted autophagy [100,114,116], achieved through modulation of PI3K/Akt/P706SK pathway [100,114] while an equally important antiproliferative mechanism was the suppression of angiogenesis [103,106,107,110,116] through downregulation of the VEGFR-2/PIEK/mTOR pathway [103], suppression of αvβ3 integrin-mediated VEGF [106], suppression of Src/FAK phosphorylation [106], and attenuation of HIF-1a-regulated VEGF [107]. Finally, regarding cancer stem cells, THD was able to selectively promote the differentiation of cancer stem cells while sparing the normal stem cells [121,122,128]. The antitumor mechanisms of action of thioridazine are summarized in Figure 5.

## 3. Atypical Antipsychotics

### 3.1. Quetiapine 

Quetiapine is a commonly prescribed atypical antipsychotic drug for treating schizophrenia, depression, and bipolar disorder. So far, only limited research has been performed regarding its potential antitumor effects. Wang et al. studied the anti-tumor effect of quetiapine in glioma stem cells isolated from the GBM GL216 cell line, which were subsequently xenografted in mice orthotopically and subcutaneously. Quetiapine suppressed glioma growth by inducing oligodendrocyte differentiation of glioma stem cells. Mechanistically, this was linked with the inhibition of the Wnt/β-catenin signaling pathway, which is involved in embryogenesis and differentiation of stem cells. Moreover, they found that quetiapine has a synergistic effect with TMZ, inhibiting the growth of TMZ-resistant glioma stem cells [129]. Recently, Saki et al. found that quetiapine increases the radiosensitivity of GBM cells in GBM mice models. In this study, the glioma cells that developed resistance in the combination of quetiapine and irradiation-expressed genes involved in cholesterol synthesis, and the addition of atorvastatin, a 3-hydroxy-3-methyl-glutaryl-coenzyme A reductase (HMG-CoA) inhibitor, enhanced the efficacy of quetiapine [130]. Moreover, epidemiological studies have shown that quetiapine reduces the risk of hepatocellular carcinoma development in patients with schizophrenia [131], while according to Lee et al., quetiapine inhibited cell proliferation and migration of Hep1 and Hep3B HCC cancer cell lines and induced their apoptosis via activation of intrinsic and extrinsic apoptotic pathways and simultaneous reduction of expression of anti-apoptotic proteins such as XIAP and survivin via inhibition of the ERK/AKT signaling pathway [132]. 

### 3.2. Olanzapine

Olanzapine is a first-line atypical antipsychotic drug that primarily acts as a DRD2 antagonist. Moreover, olanzapine inhibits various other receptors, including serotonin (5-HT2A) and muscarinic receptors, and is thus utilized for controlling chemotherapy-induced nausea and vomiting. Regarding its potential anti-cancer properties, only limited experimental studies have been performed [133]. Olanzapine inhibits the proliferation of U87MG and A172 GBM cell lines, probably by promoting apoptosis, and enhances the anti-tumor effect of TMZ. Moreover, olanzapine inhibits the anchorage-dependent growth in U87MG cell lines and inhibits cancer cell migration in the A172 GBM cell line but not in the U87MG cell line. The exact mechanism of action is currently unclear, but downregulation of β-catenin and c-jun, an oncogenic transcription factor, may be involved [134]. In vitro, Sanomachi et al. supported that olanzapine specifically inhibits various cancer stem cell lines such as lung (A549) and pancreatic (PANC-1, PSN-1) in low concentrations, around 50μΜ, without affecting normal cells and increases the efficacy of several chemotherapeutic agents possibly by decreasing the expression of survivin in these cells [135].

### 3.3. Risperidone

Risperidone (RIS) is a benzisoxazole derivative and the second atypical antipsychotic developed following clozapine. RIS received FDA approval for the treatment of schizophrenia in 1993 and, since then, has also been approved for the treatment of bipolar I disorder (2003) and autism-related irritability (2006) [136]. From a pharmacological perspective, RIS displays a profound affinity for 5-HT2A serotonin receptors and, to a lesser extent (around 10-times less), for the DRD2, while it also antagonizes the a1- and a2-adrenergic receptors. However, it does not inhibit muscarinic receptors, thus lacking anticholinergic effects [137].

Regarding its anti-cancer properties, Dilly et al. reported that RIS, combined with rumenic acid, can hamper prostate cancer cell (PC-3 cells) proliferation in vitro and slow down tumor growth in vivo in mice with PC-3 prostate cancer xenografts [138]. The latter effects were attributed to the potent inhibition of 17-β-Hydroxysteroid dehydrogenase 10 (17HSD10), a multifunctional mitochondrial enzyme, which, among many other properties, has been found to convert the very weak androgen 5alpha-androstanediol into the more potent androgen 5alpha-dihydrotestosterone (DHT), thus providing an alternative androgen synthesis pathway that can operate even in the absence of testosterone, eventually helping cancerous cells survive androgen ablation therapy [139,140].

In another study, Wang et al. investigated in vitro the interaction between several antipsychotic drugs and MCF7/MX100 (resistant breast cancer) and Caco-2 (colon carcinoma) cell lines, both overexpressing the breast cancer resistance protein (BCRP), an energy-dependent transporter of the adenosine triphosphate-binding cassette (ABC) transporter family. BCRP can be found in many different human cells, including the liver canaliculi, mucosal surfaces of the small intestine, and the colon, cardiac muscle, pancreas, adrenal cortex, thyroid and parathyroid endothelia, and others where it regulates the drug absorption, predominantly leading to drug excretion. Thus, inhibiting BCRP could potentially increase the efficacy of chemotherapy drugs in the abovementioned organs. Wang and colleagues reported that risperidone, at concentrations of 1–100μΜ, effectively inhibited the overexpression of BCRP transporter in both cell lines in a concentration-dependent manner [141]. On the same lines, Gundogdu et al. observed in vitro proliferation and migration suppression as well as dose-dependent DNA damage of MC7-cells with the application of risperidone. The exact genotoxic mechanisms were not discussed, yet the authors implied a possible role of the Dopamine D2- and 5-HT2a-associated ERK and Akt signaling pathways [142]. Finally, the in vitro study by Chen et al. in human hepatocellular carcinoma cell lines (Huh-7 and Hep G2) indicated that risperidone suppresses proliferation and invasion of these cells while it induces apoptosis by potent activation of caspase-3 [131,143].

### 3.4. Aripiprazole (ARP)

Aripiprazole (ARP) is an atypical antiphychotic drug commonly used for the treatment of schizophrenia and bipolar disorder. It has a high affinity for dopamine D2 receptors and agonizes and antagonizes, particularly the 5-HT1A and 5-HT2A, respectively [144]. Regarding its anticancer properties, Badran et al., in their in vitro studies in MCF-7 cells (breast cancer cell line), observed inhibition of cell proliferation by cell cycle arrest in subG0/G1 phase and subsequently the induction of apoptosis (downregulation of anti-apoptotic genes BCL2L1, C-myc and upregulation of pro-apoptotic genes BCL-10, BAK, active caspase-3 and caspase-9) [145]. Similarly, Lee et al., using the same cell line (MCF-7) for their in vitro studies, and also attributed the antiproliferative effect of ARP to the induction of apoptosis but, noteworthily, the D2R/AMPK activation may attenuate the latter effect [146]. In another in vitro study, ARP decreased the proliferation of pancreatic and non-small lung cancer stem cells, presumably through Wnt/β-catenin pathway inhibition. The authors also reported a significant reduction of the anti-apoptotic protein survivin as well as inhibition of P-gp pumps, both leading to reduced cells’ resistance to chemotherapeutic agents [147]. In the context of P-gp pumps, two additional in vitro studies supported that the effective P-gp inhibition by ARP increased the potency of anti-mitotic drugs when these two drugs were combined [148,149]. Finally, Kim et al., using cell culture (in vitro) and xenografted mice (in vivo), supported that ARP induced apoptosis-mediated cytotoxicity and migration suppression by direct inhibition of Src oncogenic tyrosine and therefore inhibition of the signaling cascade molecules (phosphorylated phosphatidylinositide 3-kinase, STAT3, and Akt), all involved in glioma progression [150].

### 3.5. Clozapine (CLZ)

Clozapine (CLZ) is an atypical antipsychotic drug, widely considered the most efficient drug for the treatment of resistant schizophrenia. However, its clinical utility remains limited due to undesirable side effects such as agranulocytosis, sedation, and hyperlipidosis. Clozapine extracts its antipsychotic activity by binding with high affinity to serotonin 2A receptor (5-HT2A) and with a weaker affinity to DRD2 [151]. Few studies have reported that clozapine may possess anti-cancer properties. In one in vitro study using human NSCLC cells (A549, H1299), clozapine exerted antiproliferative activity by arresting cancer cells in the G0/G1 phase and promoting autophagic cell death, presumably through inhibition of the PI3k/Akt/mTOR pathway [152]. In another study, the authors studied the effect of clozapine in melanoma cell lines both in vitro (WM35: Human primary melanoma cells, M1/15: Human metastatic melanoma cells) and in vivo (M1/15 cell line). Clozapine decreased cell proliferation in both cell lines by promoting cell cycle arrest in the G0/G1 phase and senescence (disruption of lysosomal function through enhanced activity of senescence-associated b-galactosidase, leading to terminal growth arrest), both through clozapine-induced phosphorylation of the ERK pathway, a crucial pathway for melanoma progression [153]. Finally, Shin et al. tested the effect of clozapine on PTEN-negative U-87MG glioblastoma cells in vitro. The authors observed that clozapine blocks the IP3-dependent calcium release and inhibits calcium uptake through inotropic and voltage-dependent calcium receptors, downregulating the Ca^2+^/calmodulin and subsequently the PI3K/Akt/GSK-3β pathways, inducing cell cycle arrest in the G0/G1 phase with a reduction in cyclin D1 expression and ultimately inhibiting cell cycle progression [154].

## 4. D3 Receptors’ Modulation and Cancer Treatment

Dopamine D3 receptors are located predominantly in the limbic system, both pre-and post-synaptically. Although they belong to the D2-like receptor family, the D3 receptors signaling pathways are discrepant. Animal studies suggest that D3 antagonism can effectively alleviate schizophrenia’s positive symptoms without the D2 antagonism-associated adverse effects, including extrapyramidal motor symptoms, hyperprolactinemia, anhedonia, weight gain, and metabolic dysregulations [155]. Additionally, buspirone, an azapirone anxiolytic drug, through its D3-receptor antagonistic properties, improved schizophrenia-related cognitive impairment in vivo [156], while in a double-blind randomized controlled trial in 200 patients with schizophrenia, the combination of buspirone and atypical antipsychotic drugs was more effective in improving cognitive function compared with atypical antipsychotics alone [157], postulating a positive effect of D3-receptor antagonism also in cancer-associated cognitive impairment [158]. In an in vitro study, Hussein et al. found that selective D3 receptor antagonists PG01037, NGB2904, SB277011A, and U99194 significantly enhanced the efficacy of the chemotherapeutic drugs mitoxantrone and doxorubicin in the ABCG2 over-expressing multidrug-resistant lung (H460-MX20, A549-MX10) and colon cancer (S1M1-80) cell lines by impairing the regulation of the ABCG2 transporters of those cells at non-toxic concentrations [159]. In another study, cariprazine, a D3-preferring D3/D2 receptor partial agonist used for the treatment of schizophrenia and bipolar disorder [160], was utilized in vitro in ABCG2-mediated multidrug-resistant non-small lung cancer (H460-MX20 cell line) and colon cancer cell line (S1M1-80). The authors reported that cariprazine synergistically enhanced the efficacy of the antineoplastic drug mitoxantrone by downregulating the expression of ABCG2 protein, with the exact molecular mechanism of this modulation remaining unknown [161].

## 5. Conclusions 

The antitumor effect of antipsychotic drugs is mediated through multiple distinct molecular mechanisms, and there is also a significant difference in the antitumor mechanism observed between the different antipsychotic drugs. Most experimental studies in the current topic include the older typical antipsychotics, probably due to their ability to interact with several intracellular receptors, whereas the atypical ones appear to be selective for DRD2. However, there has been an increasing research trend in the last few years, focusing on the newer atypical antipsychotics with promising results. Antipsychotic drugs also appear to increase the effect of chemotherapeutic drugs such as TMZ and doxorubicin, combating the tumor’s chemo- and radio-resistance.

So far, only three clinical trials have been performed in humans, one phase II clinical trial in chlorpromazine, one phase II clinical trial in pimozide, and one phase I clinical trial in thioridazine. The details of these clinical trials are summarized in Table 1. However, due to the increasing amount of preclinical evidence gathered in the last years, especially in typical antipsychotics, there are increasing probabilities of new clinical trials being introduced within the following 5–10 years. The most studied antipsychotic drugs regarding their anti-tumor effects are haloperidol, chlorpromazine, thioridazine, and penfluridol. On the contrary, clinical trials regarding the newer atypical antipsychotics, despite their favorable adverse effects profile, are probably not expected within the next 5 years due to the limited preclinical evidence of anti-tumor activity.

## Figures and Tables

**Table 1:** Clinical trials studying the antitumor effect of antipsychotic drugs in different types of cancer.

Cancer	Drug	Start Date	Title	NCT	Phase	Results	Estimated Completion Year/Date
Glioblastoma	Chlorpromazine	15 December 2019	Repurposing chlorpromazine for the Treatment of Glioblastoma (RACTAC)	NCT04224441	II	Not posted	15 December 2022
Metastatic Melanoma [162]	Pimozide	n/s	Phase II trial of pimozide in previously treated melanoma patients	n/a	II	17% of the evaluated patients had complete or partial response for at least 8 months	Completed (1983)
Acute myeloid leukemia	Thioridazine (THD)	July 2014	Safety study of Thioridazine in combination with Cytarabine to treat relapsed or refractory Acute Myeloid Leukemia (THORIDAL)	NCT02096289	I	A reduction up to 55% in blast levels was noted in 85% of the patients	Completed (September 2016)

## Data Availability

Not applicable.
